# APID database: redefining protein–protein interaction experimental evidences and binary interactomes

**DOI:** 10.1093/database/baz005

**Published:** 2019-01-31

**Authors:** Diego Alonso-López, Francisco J Campos-Laborie, Miguel A Gutiérrez, Luke Lambourne, Michael A Calderwood, Marc Vidal, Javier De Las Rivas

**Affiliations:** 1 Cancer Research Center (CiC-IBMCC, CSIC/USAL/IBSAL), Consejo Superior de Investigaciones Científicas and University of Salamanca, Salamanca, Spain; 2 Center for Cancer Systems Biology, Department of Cancer Biology, Dana-Farber Cancer Institute and Department of Genetics, Harvard Medical School, Boston, MA, USA

## Abstract

The collection and integration of all the known protein–protein physical interactions within a proteome framework are critical to allow proper exploration of the protein interaction networks that drive biological processes in cells at molecular level. APID Interactomes is a public resource of biological data (http://apid.dep.usal.es) that provides a comprehensive and curated collection of `protein interactomes’ for more than 1100 organisms, including 30 species with more than 500 interactions, derived from the integration of experimentally detected protein-to-protein physical interactions (PPIs). We have performed an update of APID database including a redefinition of several key properties of the PPIs to provide a more precise data integration and to avoid false duplicated records. This includes the unification of all the PPIs from five primary databases of molecular interactions (BioGRID, DIP, HPRD, IntAct and MINT), plus the information from two original systematic sources of human data and from experimentally resolved 3D structures (i.e. PDBs, Protein Data Bank files, where more than two distinct proteins have been identified). Thus, APID provides PPIs reported in published research articles (with traceable PMIDs) and detected by valid experimental interaction methods that give evidences about such protein interactions (following the `ontology and controlled vocabulary’: www.ebi.ac.uk/ols/ontologies/mi; developed by `HUPO PSI-MI’). Within this data mining framework, all interaction detection methods have been grouped into two main types: (i) `binary’ physical direct detection methods and (ii) `indirect’ methods. As a result of these redefinitions, APID provides unified protein interactomes including the specific `experimental evidences’ that support each PPI, indicating whether the interactions can be considered `binary’ (i.e. supported by at least one binary detection method) or not.

## Introduction

The experimental mapping of all molecular interactions between pairs of proteins that occur by specific biophysical contacts [i.e. protein-to-protein physical interactions (PPIs)] is an essential step to achieve a better understanding of the molecular architecture and the wiring of proteins in living cells. This biological cartography will produce a wider and more significant view when it comes close to full coverage by including data on all known proteins within a proteome (1–3). The past decade has produced a vast amount of biological data and data resources that include information about PPIs (4). Despite this large amount of PPI public data, there are many inconsistencies, duplications and misleading features that cause many false-positive records when users and researchers wish to fetch the compendium of all reported PPIs from one organism (i.e. the interactome of such an organism). The same type of problems occurs when a user wants to build a protein interaction network with all the known experimentally supported PPIs from a query list of proteins. In this work, we propose to address these problems undertaking an update of `APID Interactomes’ database (5) that includes a unification of the information from different primary databases of PPIs, removing duplicated and incomplete records. To achieve this, we have developed a systematic pipeline to collect the `experimental evidences’ that support each PPI in primary databases that curate the scientific literature and we have applied this procedure to the construction of binary protein interactomes (6). Each binary PPI should be supported by experimental interaction detection methods that are not ambiguous or undefined and that are `binary’, because they provide evidence of direct physical interactions between the tested protein pair.

## Results and discussion

### Redefining proper interaction detection methods within PSI-MI

The working group on Molecular Interactions of the HUPO Proteomics Standards Initiative (PSI-MI) has recently published an update of the standard format to enable the download and interchange of molecular interaction data (7). This standard follows a structured controlled vocabulary (www.ebi.ac.uk/ols/ontologies/mi) for the annotation of experiments concerned with PPIs. A relevant part of this vocabulary includes the `interaction detection methods’ (accessible at www.ebi.ac.uk/ols/ontologies/MI/terms?obo_id=MI:0001), which allow the identification of the methods used to detect each PPI reported in a published article, assigning a unique identifier notation to each method (like MI:000X). Most public databases and resources of PPIs follow the PSI-MI standards and apply them when creating new records, including the interactions derived from the specific research articles, which have been analyzed and curated (8). In the construction of APID (5, 9), as a meta-database that analyzes and integrates all records obtained from multiple primary databases, we also implemented PSI-MI standards and assigned `interaction detection methods’ following the nomenclature and IDs provided by PSI-MI. However, despite the exhaustive use of PSI-MI, we found several imprecise terms linked to the highest category of `interaction detection methods’ (MI:0001) since they do not correspond to a specific experimental technology applied to detect the interaction between two or more proteins. The cases of the terms are as follows: `biophysical’ (MI:0013), `inferred by author’ (MI:0363) and `inferred by curator’ (MI:0364). These terms have been included in the controlled vocabulary created by PSI-MI, but when applied to the identification of a specific interaction pair of protein A and protein B (pApB), they cannot be used as a demonstrative evidence that such interaction has been experimentally detected. Considering this observation, we had to re-evaluate all the terms and IDs included in the PSI-MI ontological vocabulary that were associated to `interaction detection method’ (MI:0001), to indicate which of them do not correspond to a proper experimental method. The results of this re-evaluation are presented in Table S1, which includes a list with all the PSI-MI terms linked to `interaction detection method’ indicating those that are not detection methods. These received the label `NotAssigned’. A total of 11 terms were included in this new category, which was called `Method_Type’.

### Defining `binary’ and `indirect’ experimental interaction detection methods

The implementation of this new category for the methods (`Method_Type’) and the re-evaluation of all the `interaction detection methods’ (Table S1) led us to a necessary reconsideration of the types of experimental technologies used to detect the physical interaction between two proteins. It is clear that some techniques are capable of detecting interactions between groups of proteins, without distinction of direct connectivity between each pair of proteins. These have been called co-complex methods (10) and a typical example is the `pull down’ technology (MI:0096). It is also clear that other experimental assays are designed to detect the direct physical interaction between specific proteins tested in pairs (11, 12). It is important to indicate that the methods of this second type generally try to prove if there is a direct contact between two specific proteins and if these two proteins can directly interact with each other in isolation (i.e. most of the times testing the proteins *in vitro*). It is also very important to emphasize that the classification of methods in those focused on identifying interactions in `protein complexes’ or those focused on identifying interactions between `protein pairs’ does not mean that a type is better, it is more accurate or it has more quality than the other. We simply report that they are different.

Considering the existence of these two different methodological approaches for experimental detection of interactions, we re-evaluated all the PSI-MI terms to assign `binary’ or `indirect’ as two major types within the new category that we defined `Method_Type’ (Table S1). Thus, our database includes this new category for each PPI. The numbers of interactions (PPIs) included in APID that correspond to each type of `interaction detection method’ or to a combination of them (i.e. only binary, only indirect, only Not-Assigned or to several of them) is presented in Table S2.

### Construction of `binary’ protein interactomes

**Figure 1:**
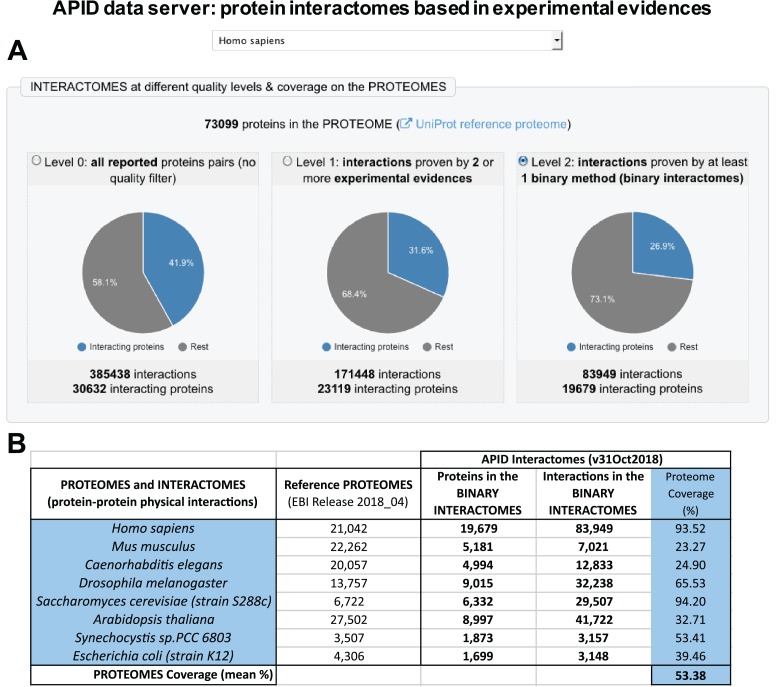
Presention of the new version of APID (Agile Protein Interactomes DataServer) that includes binary interactomes based in experimental evidences. **(A)** Panel with a view of the entry page of APID website that shows the human interactomes provided in three quality levels. **(B)** Table showing the numbers included in APID database about sizes of the binary interactomes, the corresponding reference proteomes and the coverage of the interactome on the proteome (in %) for seven model organisms and for humans.

The definition and assignment of binary methods allowed us to produce a new compendium of protein binary interactomes that are included in the updated version of APID data server (Figure 1). In this figure, it can be seen that the human binary interactome (with 83 949 PPIs) only includes ~21% of all interactions that have been reported in the literature and curated into 1 of the databases unified (which, according to our current compendium, were ~385 000; Figure 1A). This reduction in numbers can be considered appropriate because the PPIs included in the binary interactomes only are interactions detected as direct. A comparison of the number of proteins included in the human binary interactome and in the binary interactomes of 7 other model species (Figure 1B) shows that the coverage with respect to the reference proteomes is high, especially in the case of human and yeast (over 90%). The generation and analysis of binary interactomes has been a clear aim in recent studies of PPIs at a proteome scale. This has been achieved, for example, in the case of model organisms like *Escherichia coli* (13) and in the case of the human interactome (1). APID contains binary interactions for 807 organisms, including 19 species with at least 500 reported binary interactions. As far as we know, there is only one publicly accessible resource providing a compendium of binary protein interactomes that is HINT (High-quality INTeractomes) (14). This database only provides interactomes for 12 species, and it does not include all the experimental evidences for each interaction with the characteristics and criteria that we described here.

### Assignment of PSI-MI interaction detection methods by primary databases

**Figure 2:**
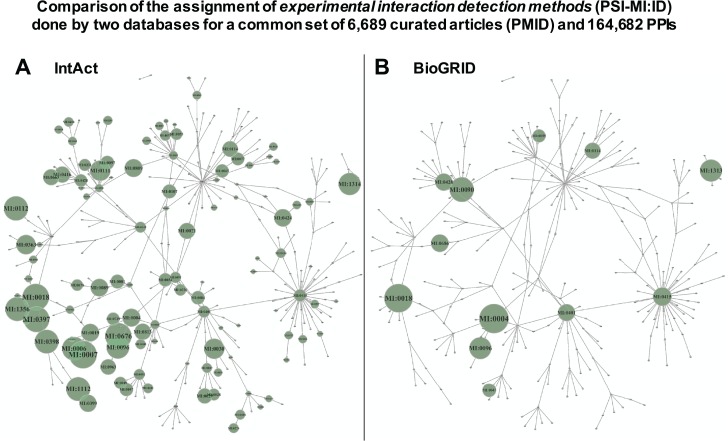
Comparison of the assignment of experimental interaction detection methods (PSI-MI:ID) done by 2 databases (IntAct and BioGRID), which included a common set of 6689 curated articles (PMID) and 164 682 PPIs. The detection methods are transformed in a network using the links between terms provided by PSI-MI. The networks include all the PSI-MI terms linked to `interaction detection method’ (271 terms, Table 1). Every term is depicted as a node in the network, and if the term is inside a green circle, it indicates that these terms are used to annotate the PPIs found by the corresponding database. **(A)** Corresponds to the network derived from IntAct data and **(B)** corresponds to the network derived from BioGRID data. The green circles are proportional to the number of times a term is used, and such circles are placed only when a term of the ontology is used.

As indicated above, we observed a great difference between the number of PPIs corresponding to the raw records derived from the source databases integrated in APID and the number of PPIs obtained in the binary interactomes (Figure 1). We investigated which could be the origin of these differences performing a deeper comparative analysis of the data derived from primary PPI databases. To do so, we choose the two larger MI databases, which are IntAct (15) and BioGRID (16), and downloaded all the PPI records that they provide in their last release of September 2018. After processing this data, we managed to identify a common set of 6689 curated articles (with specific PMID) and 164 682 PPIs that were reported by both databases in a total of 179 739 common records. This allows us to make a comparison of the assignment to experimental interaction detection methods (PSI-MI:ID) performed by each 1 of these 2 databases: they annotate to the same terms 30 310 records out of 179 739 (16.86%) and they annotate to the different terms 149 429 records out of 179 739 (83.14%). As we show below, this is an apparent difference that can be unified. However, the comparison is relevant because one of the main origins of possible false duplicated evidences for PPIs occurs when the same experiment reported in a research article is curated by two primary databases, but they assign such experiment to two different experimental detection methods (i.e. two different PSI-MI IDs). The results of the comparison are presented in Figure 2, which shows a network representation of the PSI-MI ontology corresponding to `interaction detection method’ terms used in the set of PPIs that these two resources have in common. This analysis reveals several important observations: (i) the comparison using the same research articles and the same PPIs curated by two primary databases indicates that different MI resources frequently annotate and record detection methods very differently (being, according to this comparison, IntAct the database that annotates more methods for the PPIs and into a deeper level in the ontology) and (ii) the use of different terms for the same experimental evidence derived from the same PubMed article (i.e. from the same literature) can cause a large number of duplicated records, especially when they are integrated into an unified compendium in a meta-database.

Another observation derived from the study of the PSI-MI ontology, and reflected in the networks of Figure 2 by the links shown between the terms of the `interaction detection methods’, is that the associations between the methods do not always follow a hierarchical order and, therefore, cannot be included in independent groups. In fact, when the PSI-MI ontology is displayed as a tree-like structure, it shows that there are terms linked to more than one `parent’ term. This indicates that the ontology structure is a directed acyclic graph (DAG) and that there is not a single path to reach those `child’ terms from a `parent’ term. This can be a difficulty when a database developer needs to group the terms from the deepest level following the topology of the ontology. Thus, in the PSI-MI ontology, it is not straightforward to group the terms of the lower (more specific) level, into separate, non-overlapping clusters/branches, at a higher (more general) level, based on the hierarchy. Several methods allowing the evaluation of the relationships between terms in a DAG have been proposed (mainly to compute the semantic similarity between terms) (17).

To address these observations and to unify the differences in the annotation of the same PPIs, it is essential to provide a clearer definition of what is an `experimental evidence’ and what is a `curation event’. We include these definitions in the following sections, explaining how they were developed and implemented in the new version of APID Interactomes database.

### Defining `experimental_evidence’ as distinct record in a unified PPI database

**Figure 3:**
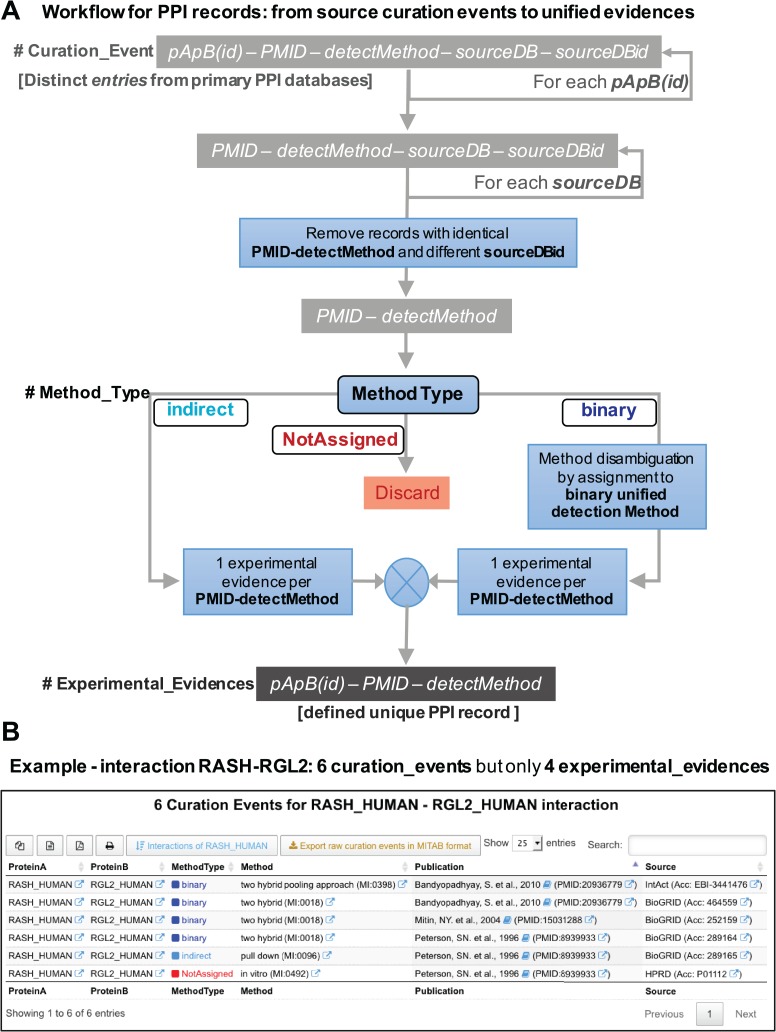
**(A)** Workflow for the PPI records that illustrates the procedure followed in the reconstruction of APID: from the original raw `curation events’ to the final unified `experimental evidences’. **(B)** View of the new data tables that APID database includes. The example corresponds to the interaction between two proteins (RASH and RGL2) and reveals that despite the existence of six original curation events, this interaction is only supported by four distinct experimental evidences.

For the development and implementation of a unified database of PPIs derived from experimental published data, we need to have a clear definition of what is a distinct or unique `record’ in such database. To achieve this, we have to define what are the essential information elements, attributes or fields that each record must have. A distinct PPI record has to include three essential and minimal elements: (i) the specific protein pair that defines the interaction (which we labeled `pApB’); (ii) the specific publication where the interaction of these two proteins have been experimentally demonstrated (which we labeled `PMID**’** because all our records must have a traceable PubMed ID associated); and (iii) the specific interaction experimental detection method (which we labeled `detectMethod’ and corresponds to a specific ID from the `HUPO PSI-MI ontology and controlled vocabulary’: www.ebi.ac.uk/ols/ontologies/mi). Therefore, any record (or tuple) in an ideal unified PPI database will correspond to a combination of these three minimal elements: `pApB’ + `PMID’ + `detectMethod’. Its implementation is critical to define unique PPI records and avoid artificial duplicates. In Figure 3A, we defined these `unique PPI records’. Avoiding duplicates involves eliminating records that do not come from accessible published studies or records that do not define well the experimental methodology used for validating a specific protein interaction. The collection of all the distinct records found for a singular protein pair (`pApB’) is what we define as the `experimental_evidences’ associated to such interaction, and it is the central building block to rebuild our unified PPI database. Thereby, the experimental evidences for a specific pair of proteins, such as for example HRAS and SOS1 proteins (HRAS_HUMAN and SOS1_HUMAN), would be generated every time that a new publication reports the molecular interaction between these two human proteins, and each experimental evidence should include the details about the specific experimental interaction detection method used to test such interaction.

### Defining `curation_event’ associated with the primary databases

After defining the minimal elements of a protein interaction record (`pApB’ + `PMID’ + `detectMethod’), it is very important to consider other attributes that should be added to this tuple (record) when there are several source databases or biological data resources that must be merged. The first attribute that has to be added when there is an integration of different databases is the one that defines each source (that we labeled `sourceDB’). In the case of APID, we have indicated that it collects and integrates all PPIs from five primary databases of molecular interactions: BioGRID, DIP, HPRD, IntAct and MINT.

In many cases, adding just the `sourceDB’ field to the records is not enough. For example, when the same interaction (defined by a record with four attributes: `pApB’ - `PMID’ - `detectMethod’ - `sourceDB’) is curated more than once by a primary database, and thus it has several IDs in the same source database. Therefore, the record also needs to have another element: the `sourceDB’ identifier that corresponds to each time a PPI record had been analyzed and re-annotated in that source database (i.e. the `sourceDBid’).

These five field records (`pApB’ + `PMID’ +`detectMethod’ + `sourceDB’ + `sourceDBid’) are what we define as `curation_event’. The exhaustive and reliable recognition of all the curation events is essential to ensure the quality of the unified database building process and to keep the traceability of all the unified PPI records.

Finally, after these definitions, it is important to underline the difference between `curation_event’ and `experimental_evidence’. The `curation_event’ is only determined by the action of a primary database to read and curate a PPI from a PubMed article and report it, and, therefore, it is independent of the experimental evidence, since a single experiment shown in an article as a demonstration of the interaction between proteins X and Y can be curated by several databases producing several `curation_events’ but only one single `experimental_evidence’. In fact, we do not count as an `evidence’ each time that one, two or three primary databases curate the same PPI from a PubMed article tested with the same method, and this is a clear difference with other PPI meta-databases.

### Re-curation into major PSI-MI terms leads to the unification of PPI records

As mentioned above, the two biggest primary databases for PPIs (IntAct and BioGRID) make a different use of the available ontology terms, which leads to differences in the curation events reported by each one for common published papers. Figure 2 showed how IntAct opts for greater depth, trying to be more specific when a PSI-MI method (`detectMethod’) is assigned to any record, while BioGRID remains in higher levels of the PSI-MI ontology. Although these differences are extended along all the ontology, we focused in binary methods to improve the quality of the metrics supporting each single interaction in the generation of the binary interactomes. Therefore, we propose a reunification of binary PSI-MI terms changing original assigned terms or methods by `binary unified detection methods’, which can be considered as `meta-methods’ and are defined as the most representative PSI-MI terms within a group of related PSI-MI terms. These pivotal unified methods have been chosen considering the common characteristics of the technology with others and the simplicity to understand by the user. Table S1 includes a column (called `MI:ID of binary unified detection method’) that describes the chosen PSI-MI ontology terms. This disambiguation step is placed in the APID procedure to define a unique PPI record, being applied only to binary PSI-MI terms.

### Implementation of these new definitions in new APID database

Figure 3A depicts a workflow that illustrates and explains the procedure followed by the APID algorithm to extract all the experimental evidences from a collection of curation events reporting the interaction between pApB in several primary databases. It is relevant to mention that the last two elements of the `curation events’ should be transparent to the users of a unified PPI database, since they do not affect the defined `experimental_evidences’.

Figure 3B presents a view of the new data tables that APID database includes. The example corresponds to the interaction between proteins RASH and RGL2 and reveals that despite the existence of six original curation events, this interaction is supported only by four distinct experimental evidences after the application of the previously described workflow.

To validate our proposal, we measured the ability of this reunification step to solve curation mismatches among IntAct and BioGrid. Based on the same 6689 common papers, there were 86 382 curation events (48.05% of the total) including at least 1 binary `detectMethod’. Out of these records, 35 673 curation events (41.29%) show differences between IntAct and BioGrid. After the reunification step into meta-terms, we matched registers from both primary databases obtaining an almost total agreement (99.91%).

## Conclusions

The analysis of the essential elements necessary to produce unique records for a PPI database led us to redefine what is a single `experimental evidence’ and what is a `curation event’. These redefinitions allow us to reconstruct the protein interactomes included in APID database, based on the integration of currently known data derived from multiple primary sources. Thus, the new version of the APID database presented in this article only counts unique `experimental evidences’ for the protein interactions included in the interactomes provided. In addition, the identification of binary interaction detection methods within the list of registered PSI-MI ontology terms allows us to generate a new compendium of `binary interactomes’ for multiple species, with reliable metrics to weight every interaction included in each of those interactomes.

## Funding

Instituto de Salud Carlos III (ISCiii, Spain: PI18/00591 and AC14/00024); FEDER program of the European Union; and European Project H2020 ArrestAD (ID: 737390, H2020-FETOPEN-1-2016-2017).

*Conflict of interest*. None declared.

## Supplementary Material

Supplementary Table 1

Supplementary Table 2

## Notes

**Database URL:** http://apid.dep.usal.es
